# Which Genes in a Typical Intertidal Seagrass (*Zostera japonica*) Indicate Copper-, Lead-, and Cadmium Pollution?

**DOI:** 10.3389/fpls.2018.01545

**Published:** 2018-10-24

**Authors:** Haiying Lin, Tao Sun, Yi Zhou, Ruiting Gu, Xiaomei Zhang, Wei Yang

**Affiliations:** ^1^State Key Laboratory of Water Environment Simulation, School of Environment, Beijing Normal University, Beijing, China; ^2^Key Laboratory of Marine Ecology and Environmental Sciences, Institute of Oceanology, Chinese Academy of Sciences, Qingdao, China

**Keywords:** genes, transcriptomic analysis, heavy metals, seagrass, Zostera japonica

## Abstract

Healthy seagrasses are considered a prime indicator of estuarine and coastal ecosystem function; however, as the only group of flowering plants recolonizing the sea, seagrasses are frequently exposed to anthropogenic heavy metal pollutants, which are associated with high levels of molecular damage. To determine whether biologically relevant concentrations of heavy metals cause systematic alterations in RNA expression patterns, we performed a gene expression study using transcriptome analyses (RNA-seq). We exposed the typical intertidal seagrass *Zostera japonica* to 0 and 50 μM of copper (Cu), lead (Pb), and cadmium (Cd) under laboratory conditions. A total of 18,266 differentially expressed genes (DEGs) were identified, of which 2001 co-expressed genes directly related by Cu, Pb, and Cd stress. We also examined the effects of short-term heavy metal Cu, Pb, and Cd pulses on the accumulation of metals in *Z. japonica* and showed metal concentrations were higher in the shoots than in roots. Twelve differentially expressed genes were further analyzed for expression differences using real-time quantitative polymerase chain reaction (RT-qPCR). Our data suggest that as coastal seawater pollution worsens, the sensitive genes identified in this study may be useful biomarkers of sublethal effects and provide fundamental information for *Z. japonica* resistant gene engineering.

## Introduction

Seagrasses are a polyphyletic and unique group of monocotyledonous angiosperms that are capable of living in both marine and estuarine waters (Larkum et al., 2006), and provide a wide variety of important ecological services (Costanza et al., 1997; Barbier et al., 2011; Fourqurean et al., 2012). However, seagrass meadows are disappearing at an alarming rate around the world due to coastal development and global change (Orth et al., 2006; Waycott et al., 2009; Short et al., 2011). As the first barrier against the sea, seagrass ecosystems are directly exposed to various anthropogenic stresses from boat traffic, dredging, land reclamation, and industrial and domestic runoff (Orth et al., 2006). Therefore, the effect that humanity has on seagrass meadows globally is devastating. Aquatic ecosystems are more sensitive to heavy metal pollutants than terrestrial ecosystems, and heavy metals originating from natural and anthropogenic sources contaminate seagrass meadows (Halpern et al., 2008). Important sources of heavy metal input into the marine environment include atmospheric deposition, domestic wastewater and industrial discharges, fertilizers, pesticides, and municipal sewage sludge (Heim and Schwarzbauer, 2013; Bazzano et al., 2014). Heavy metal suspensions can affect seagrass via the reduction of light availability, and they can hamper water movement, which enhances oxygen consumption and the enrichment of organic products and nutrients that cause environmental stress (Papathanasiou et al., 2015). Moreover, heavy metals are a source of pollution, and their alkaloid molecules have cytotoxic effects that negatively affect the growth of seagrasses. Accumulation of Cu, Cd, and Pb in the sediment of seagrass ecosystems was examined using *Zostera marina* were also invetigated, the results indicated that *Zostera marina* leaves can cause Cu and Pb accumulation in sediments in seagrass ecosystems, but Cd concentration were significantly lower in the surface sediment than in the leaves (Hosokawa et al., 2016). A thorough understanding of the molecular mechanisms and heavy metal uptake processes associated with seagrasses has not yet been established. However, the recent development of molecular research for different seagrass species (e.g., *Zostera marina* and *Posidonia oceanica*) has greatly facilitated the investigation of differentially expressed genes (DEGs) affected by environmental changes, including heat stress, varied light conditions, depth, temperature stress, salinity, and pH effects (Reusch et al., 2008; Bergmann et al., 2010; Bruno et al., 2010; Winters et al., 2011; Serra et al., 2012; Dattolo et al., 2013, 2014). For *Zostera marina*, three genes for metallothionein designated as *Mtlp-Zm2.1, Mtlp-Zm2.2*,and *Mtlp-Zm3*.(Accession numbers: JG699194, JG699014, and JG699205, respectively) in this seagrass that was identified (Kong et al., 2013) Regarding dwarf *Zostera* species, only a few studies examined *Nanozostera noltii* and *Zostera notili* under simulated heat wave and light attenuation conditions, which mimicked global warming patterns (Massa et al., 2011; Gu et al., 2012; Franssen et al., 2014).

The seagrass *Z. japonica* belongs to the family Zosteraceae, and it inhabits intertidal sandy and muddy bottoms at water depths of approximately <1 m. The species is distributed within the temperate and subtropical regions of the northern Pacific Coast, i.e., the east coast of Asia and the west coast of North America, and it is native to the coasts of eastern Asia, from Russia to Vietnam (Short et al., 2007). Natural *Z. japonica* populations in Asia have not been immune to the alarming decline caused by coastal development and anthropogenic pollution (Lee, 1997; Lee et al., 2004; Abe et al., 2009; Zhang et al., 2015b). Recent surveys in China found that *Z. japonica* habitat was divided into two main areas, northern and southern China (Zhang et al., 2015a,b; Lin et al., 2016a).

The molecular technologies developed over the past 20 years have undoubtedly provided insights into seagrass genetics and their adaptability to environmental stress, especially the analysis of gene expression, which is a powerful approach that can determine how organisms respond to particular abiotic conditions (Hou et al., 2015; Hossain et al., 2016). Recently developed RNA deep-sequencing technology provides a platform to measure differences in gene expression, which is more sensitive than traditional microarray hybridization techniques (Wilhelm and Landry, 2009; Saminathan et al., 2015a). This new method dramatically improves the procedures used to identify DEGs (Cloonan et al., 2008; Garber et al., 2011). The transcriptome sequencing method (RNA-seq) generates absolute information, rather than relative gene expression measurements, and it is more sensitive when detecting low-expressed transcripts. For seagrass, transcriptome-based analysis of marine vegetation has provided novel insights into their adaptation to the highly dynamic environmental conditions typically observed in marine ecosystems (Franssen et al., 2011; Dattolo et al., 2013).

In the present study, RNA-seq was employed to explore the mechanisms associated with metal stress in *Z. japonica*. Modern transcriptome methodologies can quantify the expression of most genes in an organism, based on the RNA transcript levels observed in response to a pollutant condition relative to the levels observed under normal physiological conditions (Leng et al., 2015). Comparisons of the effects of other specific conditions can reveal degrees of similarity or difference. The RNA-seq approach generates a vast inventory of gene transcripts using massive parallel DNA sequencing technologies, bioinformatics, and sequence databases, and it can identify novel exons and splice junctions. Therefore, although transcriptome profiling does not reveal changes associated with effects at the translational, posttranslational, cell biological or organismal levels, it does provide substantial and detailed information about toxicological responses to different stresses (Simon et al., 2013).

*Zostera japonica* absorbs and accumulates metals from sediments in its organs and tissues, which determines metal bioavailability in the marine ecosystem (Lin et al., 2016a). Lead (Pb), arsenic (As), cadmium (Cd), chromium (Cr), and copper (Cu) concentrations in surrounding environments present potential risks to seagrass habitats in the Yellow River Estuary (Lin et al., 2016a). In our previous *Z. japonica* study, we found that in the Yellow River habitat, heavy metals were 1.00–2.03 times higher in seagrass-rooted sediment than in the adjacent non-seagrass sediment. Moreover, Pb levels in *Z. japonica* samples from Huiquan Bay exhibited stronger anthropogenic impacts than those from the Yellow River Estuary (Lin et al., 2016b). Until now, little knowledge of the *Z. japonica* response to environmentally relevant medium metal levels, which causes sublethal metal toxicity, has been available. Therefore, the present study aimed to examine the following: (i) *Z. japonica* metal uptake and seagrass responses to it in terms of uptake ability; (ii) how feedback from biological mechanisms affects the transcriptome analysis; and (iii) detailed changes in co-expressed genes as the genetic markers of meadow resilience in response to a heavy metal disturbance event. In this study, from the relatively high-risk metals in these two *Z. japonica* habitats, the following three metals were selected as candidates: Cu, an essential macronutrient for plant growth and development; Pb, a non-essential element; and Cd.

## Materials and methods

### Sampling locations and collection

*Zostera japonica* plants were collected from the intertidal seagrass beds (at low tide sites) from Huiquan Bay in Qingdao (E 120.21°, N 36.05°) in northern China in early April of 2015, and surface seawater samples from the sites were collected. Although the specific causes of the recent dramatic seagrass decline in Huiquan Bay are unknown, anthropogenic habitat deterioration (e.g., fishing) and environmental water contamination (e.g., wastewater discharge and port transportation) during the past decades of the twentieth century may be responsible. To ensure homogeneity, samples were vegetative propagated from a single genotype using stolon and rhizomes, and young new shoots were chosen for pot experiments.

### Experimental conditions

Following a 24 h acclimation period, *Z. japonica* plants were planted in a hydroponic system in a growth chamber containing natural seawater from Huiquan Bay. The condition of the seawater was maintained as follows: salinity 32.3–33.6; temperature 19.3–20.4°C; dissolved oxygen (DO) concentration of 4.94–5.49 mg/L; oxidation-reduction potential (ORP) 44.1–50.6 mV; and a pH 7.84–8.01, natural light. At the start of the experiment, seawater used in the four sample replicates was supplemented with different concentrations (0 and 50 μM) of Cu, Pb, and Cd. Seagrass tissues were sampled seven days after the start of the treatments for RNA-seq (three replicates each) and qPCR (three replicates each) analyses; four replicates each for metal accumulation analyses.

### Experiment 1. quantification of three metals in seagrass tissues

Seagrass samples (four replicates) from all treatments at 0.5, 1, 2, 4, and 7 days were harvested and analyzed for metals' content. Details see the Methods section (Lin et al., 2016b).

### Experiment 2. transcriptomic profiling analysis

#### RNA extraction, cDNA library construction, sequencing, and assembly

After 7 days of metal treatment, three control and three treatment plants were selected for Illumina sequencing (Allwegene Technology Inc., Beijing, China). In each case, the whole plant, including roots and shoots, was sampled as a single replicate. Following harvest, tissues were immediately frozen in liquid nitrogCen, and were stored at −80°C until use. The total RNA of each sample was extracted using an E.Z.N.A. Plant RNA Kit (OMEGA Bio-Tek, United States), according to the manufacturers' protocol. RNA from the four samples were used separately to construct the cDNA libraries, which had fragment lengths of 250 bp (±25 bp). Paired-end sequencing was then performed using the Illumina HiSeq™ 2000 sequencing platform (Illumina, San Diego, CA, United States), according to the manufacturer's instructions, and reads from each library were assembled. After trimming adapter sequences and filtering low quality reads, the clean reads were used to construct a *de novo* assembly of the QYZM transcriptome using Trinity software (Garber et al., 2011).

#### RNA-Seq, annotation, and functional enrichment analysis

Gene expression levels were measured as a unit of the expected number of fragments per kb of transcript sequence per million bp sequenced (FPKM) (Trapnell et al., 2010). DESeq software was used to identify differentially expressed genes (Thomsen et al., 2010). No genomic reference is available for this seagrass species (*Z. japonica*). For the related species *Zostera marina*, genomic information has been reported (Olsen et al., 2016), but the whole genome annotation is not complete yet. Therefore, a transcriptomic reference was used for reads that were annotated based on seven public databases [NCBI nucleotide sequences (NT), Gene Ontology, Kyoto Encyclopedia of Genes and Genomes, clusters of Orthologous Groups of proteins, Clusters of Orthologous Groups of proteins (COG), euKaryotic Ortholog Groups (KOG), and Protein family (PFAM) databases] and its sister species *Zostera marina* genomic (Figure S4 in **Supporting Information**). A total of 74,204 unigenes (53.26%) were successfully annotated.

Gene sets composed of DEGs, between defined groups of libraries, were tested for the enrichment of functional categories. A Blast cutoff e-value of 1 × 10^−5^ was used to search the NR proteins in the NCBI database, and it was also used to annotate the transcripts with corresponding GO terms. The Gene Ontology (GO) enrichment assignment analysis of the DEGs was implemented using the GOseq R package based the Wallenius non-central hyper-geometric distribution (Young et al., 2010), which can adjust for gene length bias in DEGs (http://www.geneontology.org/). In addition to GO analysis, Kyoto Encyclopedia of Genes and Genomes (KEGG) pathway analyses were also conducted. KEGG (Kanehisa et al., 2008) is a database resource designed to aid in the understanding of high-level functions and utilities of biological systems (http://www.genome.jp/kegg/). Similarly, mapping against KEGG genes involved the use of KOBAS v. 2.0 software (Xie et al., 2011) using a blast cutoff e-value of 1 × 10^−5^, and the significant enrichment threshold value is *padj* < 0.05 or corrected *padj* < 0.05. In addition, KO was used to perform orthologous analysis between different species, and the annotated database is based on the KEGG. Additionally, we did ORF prediction using orffinder (https://www.ncbi.nlm.nih.gov/orffinder/) and aligned them with the *Zostera marina* genome.

### Experiment 3. RT-qPCR validation of differential gene expression

Primers used to amplify differentially expressed RNA transcripts were designed for RT-qPCR analyses using Primer Express v. 3.0.1 software (Applied Biosystems, Foster City, CA, United States) (Table S4 in **Supporting Information**). RT-qPCR procedures outlined by Saminathan (Saminathan et al., 2015b) were used. The relative quantification based on three biological replications was normalized to the 18SrRNA reference gene and calculated using the method described by Livak et al Livak and Schmittgen (2001).

## Results

### Properties of metal content in *Z. japonica* tissues

**Figure 1:**
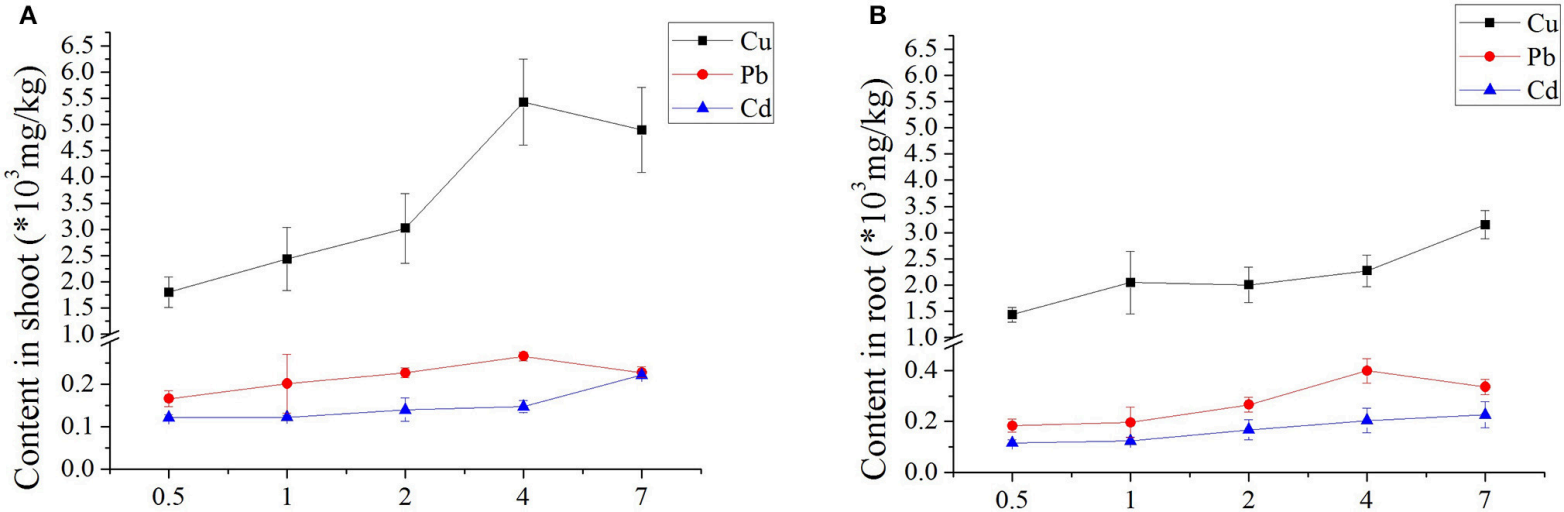
Accumulation of three heavy metals in *Zostera japonica* root and shoot tissues after 0.5–7 days of exposure. The error bars indicate the mean ± SD (*n* = 4).

The heavy metal uptake in both above- and below-ground seagrass tissues increased with increasing concentrations of Cu, Cd, and Pb for all treatments (Figure 1). Initial days of Cd, and Pb showed no significant differences in metal uptake; and obvious differences only appeared only at Cu with increasing days; and detailed was higher than uptake in the roots. Regarding the Pb treatments, varied days (0.5–7 days) resulted in minimal variation between shoot and root. Furthermore, when exposed to Cd, the seagrass tissues accumulated more Cd as the treatment concentration and duration of exposure increased (Figure 1). Therefore, the typically treatment was Cu-contained, with higher uptake concentrations in both above- and below-ground seagrass tissues, and the uptake of the shoot from low to high concentrations was higher than that observed in the root (Figure 1).

### RNA-Seq and transcriptome analyses

Some gene regulation occurs in response poor conditions to which seagrasses are exposed. The quality and length distribution details associated with the raw sequence reads and clean reads were presented in Table S2 and Figure S1 (**Supporting Information**). Briefly, all 14.47 G of clean sequencing reads, derived from four libraries, were *de novo* assembled using the Trinity software. Gene expression levels were estimated using RSEM (Li and Dewey, 2011) for each sample. Clean data were initially mapped back onto the transcriptome, and the read count for each gene was then obtained from the mapping results. A full comparison of the DEGs for each treatment and a full list of the DEG levels and the associated FPKM density distribution for each treatment were shown in Figure S2 (**Supporting Information**).

### Characteristic gene expression profiles induced by three heavy metals

**Figure 2:**
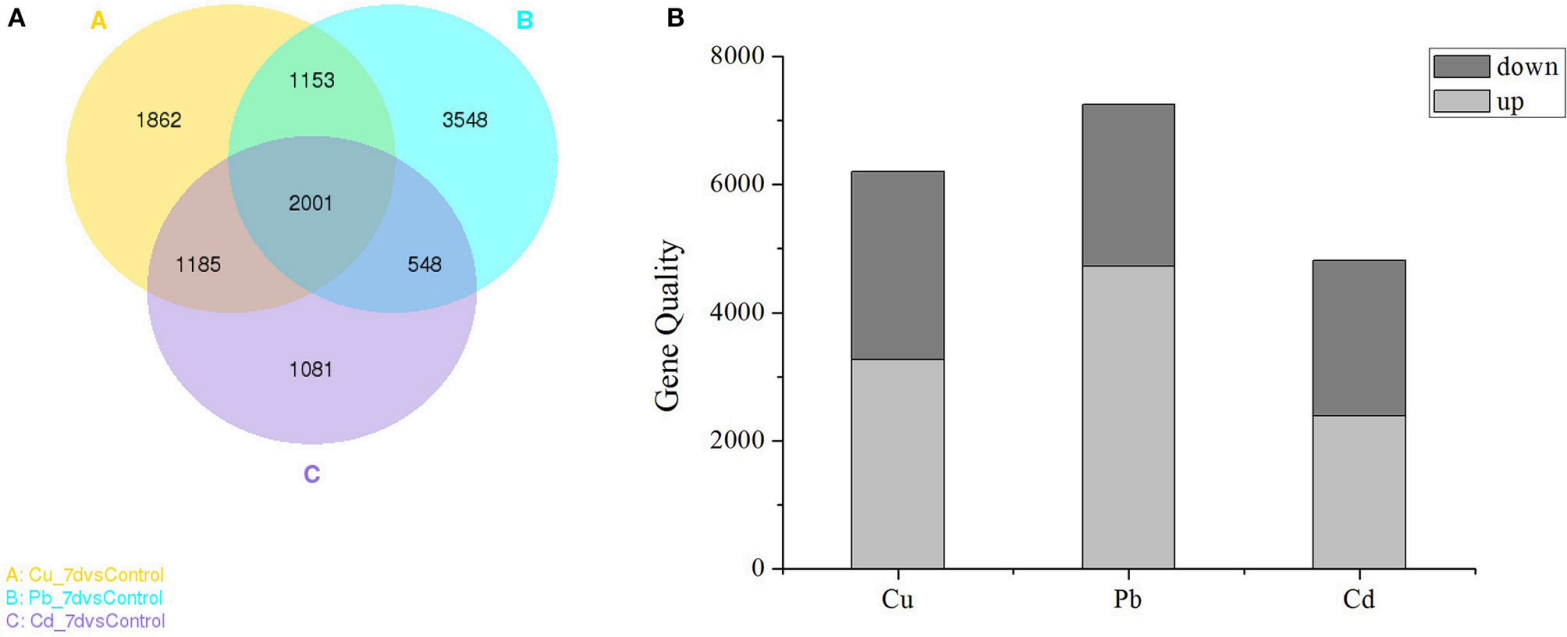
**(A)** Venn diagram showing the number of differentially expressed genes (DEGs) in each treatment (Cu, Pb, and Cd) that were significantly different from the control. **(B)** Numbers of DEGs found in the three metal treatments. The “*padj* < 0.05” criterion was used as a threshold to determine the significance of differential gene expression.

As shown in the Venn diagram (Figure 2A), the overlapping regions (2001 individual genes) represent DEGs that were common in multiple treatments, and the non-overlapping regions represent DEGs that were unique to single treatments (Figure 2). In Cu, Pb, and Cd treatments, 1,862 (10.19%), 3,548 (19.42%), and 1,081 (5.92%) genes were differentially expressed (*padj* <0.05) (Figure 2A), respectively. Pb exhibited more DEGs than Cu and Cd, and 2001 (10.95%) genes were co-expressed in all three treatments. Furthermore, statistical analyses revealed that exposure to Cu, Pb, and Cd treatments caused the observed differential expression (*padj* < 0.05) of 6201 genes (3266 upregulated and 2935 downregulated), 7250 genes (4729 upregulated and 2521 downregulated), and 4815 genes (2388 upregulated and 2427 downregulated), respectively (Figure 2B). There were more upregulated than downregulated genes in all three metal treatments, which indicated that gene activation/upregulation was a common feature of the three metal treatments (Figure 2B and Table S3 in **Supporting Information**). Together, these results suggested that exposure to heavy metals at 50 μM strongly affected gene expression in *Z. japonica*.

To visualize the differences in the gene expression profiles for all three heavy metal treatments, the DEGs were analyzed using a one-way ANOVA, and the most significant genes (*padj* < 0.05) were subjected to clustering analyses. As shown in Figure S3 (**Supporting Information**), the gene expression profiles induced by Cu, Pb, and Cd were divided into two subclusters. The right subcluster consisted of samples exposed to control, Cd and Cu-spiked solutions, and the left subcluster included samples exposed to Pb treatments. These results indicated that characteristic gene expression profiles can be used to discriminate each of the metal treatment groups from the Pb and controls, and they can discriminate all three metal treatments from each other.

### Validation of the DEGs expression differences

**Figure 3:**
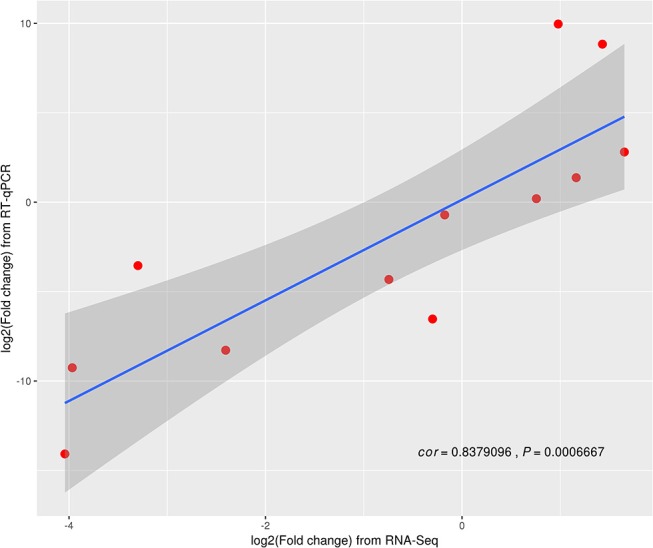
Differentially expressed genes (DEGs) validated by RT-qPCR.

Twelve genes in random (three replicates) were examined and further analyzed using RT-qPCR (Table S4 in **Supporting Information**). Based on the *Z. japonica* gene expression levels as measured by RT-qPCR, compared to those measured using RNA-seq methods, the results indicate that all 12 genes exhibited different expression levels in three treatments (*p* < 0.001; Figure 3). A highly significant correlation (*R*^2^ = 0.84) was observed between the RNA-seq and RT-qPCR data sets. Moreover, the trend associated with expression changes of these genes based on qPCR was the same as that detected by the RNA-seq method, which confirmed that the RNA-seq method generated reliable expression data.

### Functional and pathway result

An enrichment analysis was used to further identify the genetic markers and biological processes influenced by Cu, Pb, and Cd treatments. The results indicated that 1,862 Cu-specific, 3,548 Pb-specific, and 1,081 Cd-specific genes were categorized into 2,129, 2,141, and 2,033 biological processes, respectively.

**Figure 4:**
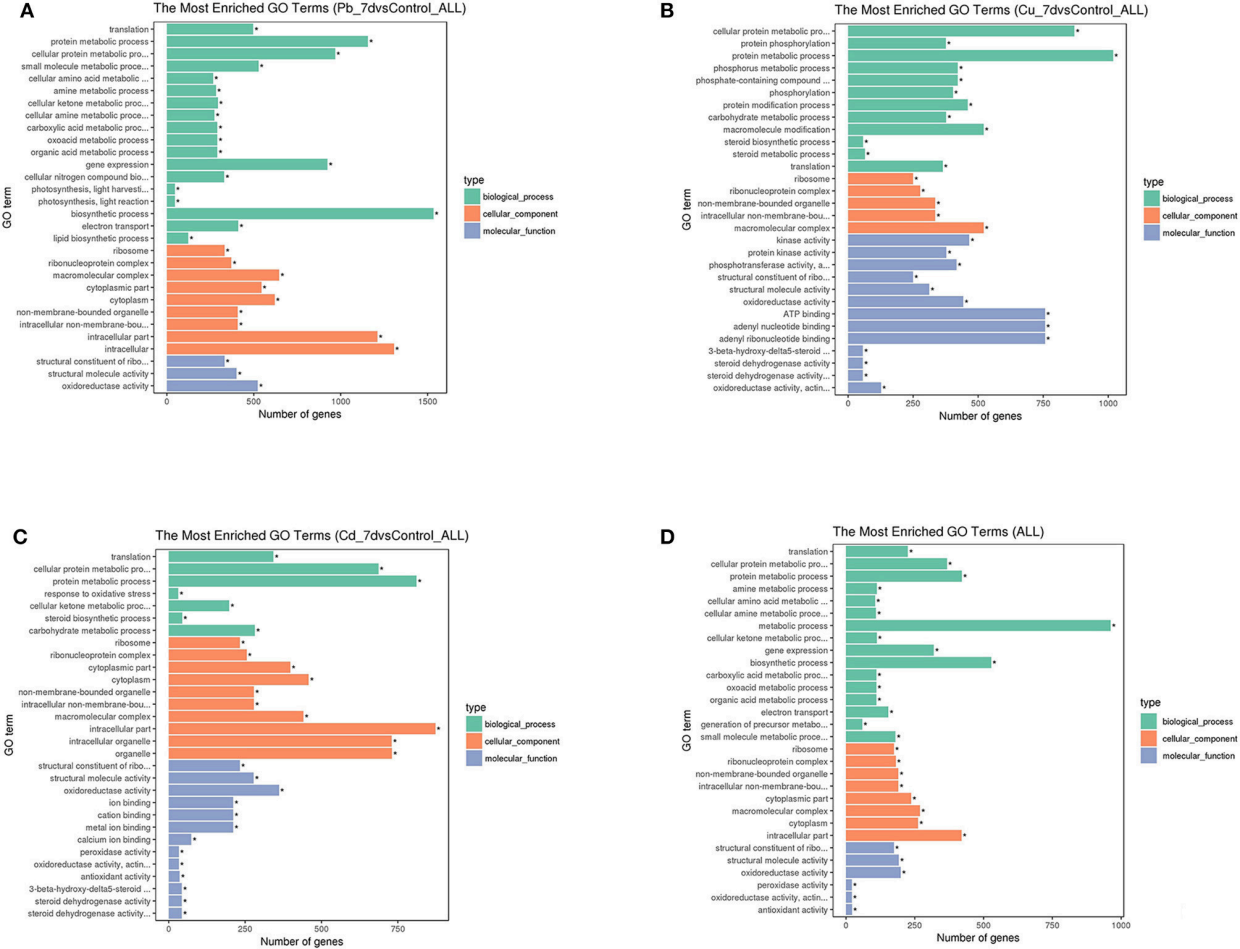
Histogram of three major gene ontology (GO) slim categories associated with molecular function (*padj* < 0.05), biological processes, and cellular components for genes with differential expression for each metal [Pb **(A)**, Cu **(B)**, and Cd **(C)**] treatment and co-expressed genes under all three conditions **(D)**. The x-axis indicates different subcategories, and the y-axis denotes the absolute numbers of gene for all terms in each category, *means significantly enriched. **(A)** Histogram of three major gene ontology (GO) slim categories associated with molecular function (*padj* < 0.05), biological processes, and cellular components for genes with differential expression for Pb treatment. **(B)** Histogram of three major gene ontology (GO) slim categories associated with molecular function (padj < 0.05), biological processes, and cellular components for genes with differential expression for Cu treatment. **(C)** Histogram of three major gene ontology (GO) slim categories associated with molecular function (*padj* < 0.05), biological processes, and cellular components for genes with differential expression for Cd treatment. **(D)** Histogram of three major gene ontology (GO) slim categories associated with molecular function (*padj* < 0.05), biological processes, and cellular components for genes with differential expression for co-expressed genes under all three conditions.

GO enrichment analyses were used to further assess the DEG sets, and the results were summarized with GO slim terms (Figure 4). Regarding the molecular function category, structural constituent of ribosome associated with Cd and Pb exposure, kinase activity associated with Cu exposure were abundant terms, and the abundance of the general term “antioxidant activity” was ranked as follows: Pb > Cu > Cd. (Figure 4A–C) The “translation,” “protein metabolic process,” and “cellular protein metabolic process” terms were the top biological process terms associated with the Cu, Pb, and Cd treatments (Figure 4D). Moreover, “response to oxidative stress” term was also common; thus, the change in gene expression was likely due to stress caused by various abnormal factors associated with the metal stress conditions. The cellular component terms “cellular component,” “cell part” and “cell” were among the top abundant terms, which suggested the occurrence of changes in the activity of cells and the sequestration of heavy metal compounds in the vacuoles. In general, the abundance of these terms was ranked as follows: Pb >Cd >Cu. Furthermore, despite an increased number of genes/transcripts associated with Pb treatments, the results indicated that increased accumulation of metals under Pb conditions might result from a greater number of genes/transcripts in the transporter category as compared to those associated with Cd and Cu. These genes/transcripts are transporters, which play key roles in sequestering heavy metals into vacuoles without having detrimental effects on physiological homeostasis.

## Discussion

### Metal associated transfer ability in *Z. japonica* tissues

Our results indicated that the Cu concentration was higher in shoots than in roots, which possibly reflects the effective Cu ion exclusion mechanisms in the roots. The key mechanism of metal accumulation is the release of chelators from plant roots into the rhizosphere, which increases the solubility of metals in terrestrial plants. In aquatic plants, chelators might be released from both the shoots and roots, *Z. japonica* seemed to be a better hyperaccumulator of Cu, Pb, and Cd. *Zostera.marina* leaves,which can be a source of Cu, Pb, and Cd, and the leaves can cause Cu and Pb accumulation in sediments in seagrass ecosystems, but Cd were significantly lower in the surface sediment than in the leaves (Hosokawa et al., 2016). Using our cultivation conditions in seawater, high metal tolerance and hyperaccumulation in *Z. japonica* might be mediated by metal-pumps such as heavy-metal ATPase transporters (HMA) (Papoyan and Kochian, 2004), NAC TFs (N-terminal DNA binding domain transcription factors) (Liu et al., 2014), natural resistance-associated macrophage proteins (NRAMPs), and/or ABC transporters (Curie et al., 2000; Vert et al., 2002; Verret et al., 2004). ATPases as far as we known, which pump out metals using ATP to drive the reaction, and proton antiports, which use the proton gradient to pump metals across the cell membrane in the previous research (Nies, 2003).

The translocation factor (TF) was used to evaluate the ability of seagrass to transfer metals from the root to tissues above.

(1) $$\begin{array}{r} \text{TF}=\frac{\text{metals in shoot}}{\text{metals in root}} \end{array}$$

If the high enrichment plant transfer coefficient is >0.5 (Baker, 1981), then Cu, Pb, and Cd are all at high enrichment. This result likely occurs because aquatic plant leaves direct the absorption of heavy metals in the water. Results shows the increased Cu concentrations and transfer coefficients, which initially decreased before rising. Moreover, on the fourth day, TF peaked at the 50-μM concentration, and the value increased by 288% compared to the control concentration. Therefore, the order of heavy metal transfer sensitivity in *Z. japonica* was Cu > Cd > Pb (Table S1 in **Supporting Information**). The similar result also found in *Zostera.marina* leaves, which can be a source of Cu and Pb cycling in eelgrass ecosystems by accumulating after shedding and decomposing. But Cd were significantly lower in the surface sediment than in the leaves, the Cd concentrations that decreased during leaf decomposition increased in the surface sediment because of leaves that still contained higher Cd (Lee et al., 2004).

### Functional and pathway analyses

Our results indicated that the transcriptomes associated with the three metals dominated almost all of pathway categories, including Ribosome, Biosynthesis of amino acids, Carbon metabolism, Carbon fixation in photosynthetic organisms, Alanine, aspartate, and glutamate, Glyoxylate and dicarboxylate metabolism, etc. (Table 1). Metabolomic investigations have been conducted on terrestrial plant abiotic stress, and plant–pathogen interactions were recently reported (Kaplan et al., 2004; Allwood et al., 2008; Shulaev et al., 2008). Generally, the contributions of metabolic responses were found to respond more quickly to abiotic stress, and changes in transcript abundance from different biosynthetic pathways correlated with changing metabolite levels (Chechik et al., 2008; Ralser et al., 2009).

**Table 1:** Functional classification of pathway-related genes (*q* < 0.05) based on the KEGG analyses.

**KEGG pathway category**	**Cu**	**Pb**	**Cd**
Ribosome	168	212	160
Biosynthesis of amino acids	143	165	125
Carbon metabolism	160	165	136
Carbon fixation in photosynthetic organisms	60	52	47
Alanine, aspartate and glutamate	32	34	32
Glyoxylate and dicarboxylate metabolism	44	47	37
Photosynthesis–antenna proteins	19	0	18
Glycolysis/Gluconeogenesis	71	0	60
Citrate cycle (TCA cycle)	39	0	31
Phenylalanine, tyrosine and tryptophan biosynthesis	0	39	32
Porphyrin and chlorophyll metabolism	0	39	26
	**Cu-specific**		
Isoquinoline alkaloid biosynthesis	15	0	0
Pyruvate metabolism	46	0	0
Phenylalanine metabolism	22	0	0
Arginine biosynthesis	20	0	0
		**Pb-specific**	
Proteasome	0	43	0
Glycine, serine and threonine metabolism	0	48	0
Valine, leucine and isoleucine biosynthesis	0	16	0
2-Oxocarboxylic acid metabolism	0	37	0
Ribosome biogenesis in eukaryotes	0	52	0
			**Cd-specific**
Pentose phosphate pathway	0	0	42
Starch and sucrose metabolism	0	0	115
Pentose and glucuronate interconversions	0	0	55

### Which genes in a typical intertidal seagrass (*zostera japonica*) indicate copper-, lead-, and cadmium pollution?

To test the hypothesis that the RNA-seq analysis is of *Z. japonica* is an effective tool and to explore the effects of the three metals on *Z. japonica* gene expression profiles, gene expression data were analyzed for co-expressed genes and genes with specific differences (i.e., Cu-specific, Pb-specific, and Cd-specific) under seagrass stress response was discussed and analyzed.

**Figure 5:**
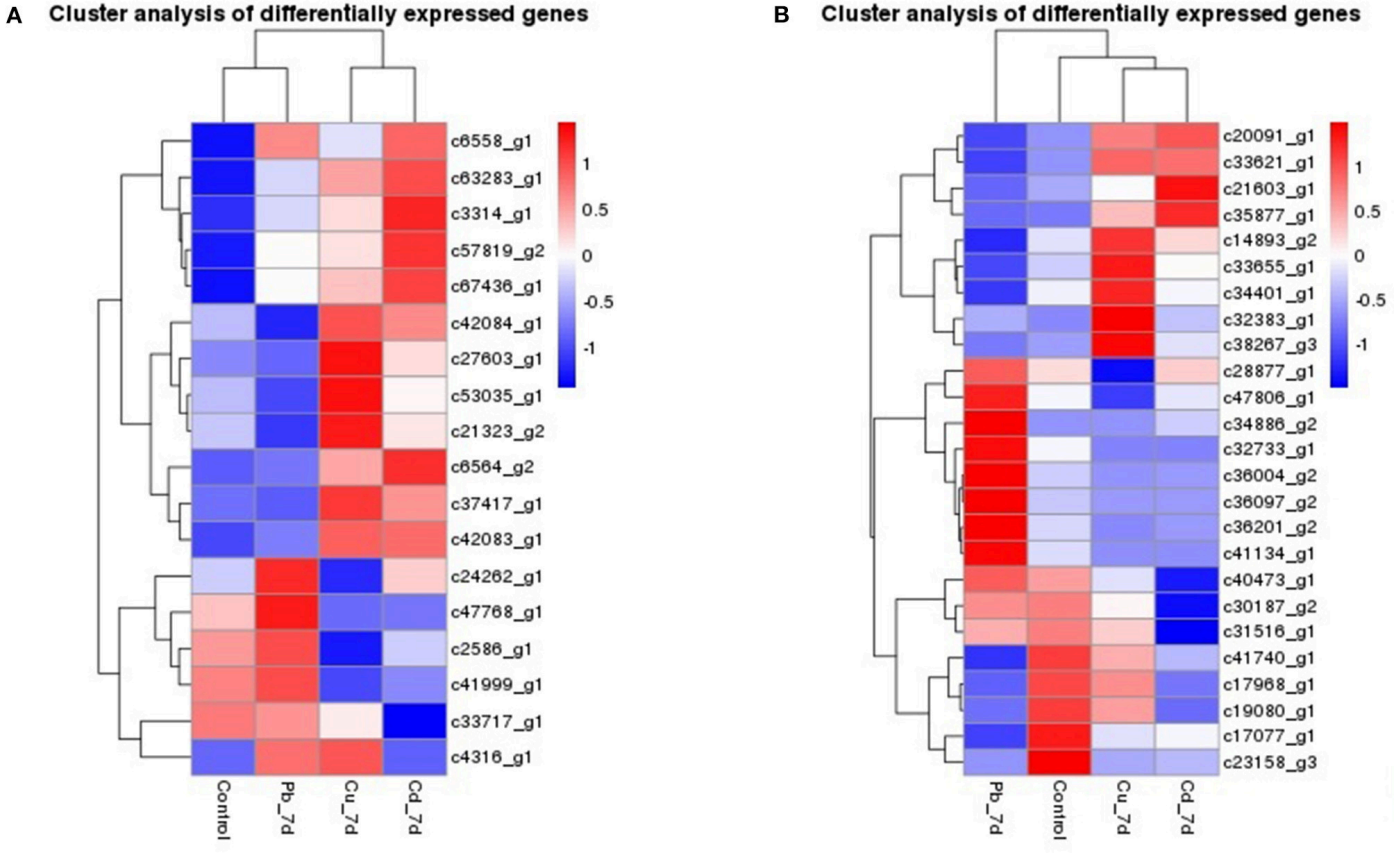
Hierarchical clustering of the genes in *Zostera japonica* under Cu, Pb, and Cd exposure. **(A)** Hierarchical clustering of the genes that were co-expressed in *Zostera japonica* under Cu, Pb, and Cd exposure. **(B)** Hierarchical clustering of the genes that were specific-expressed in *Zostera japonica* under Cu, Pb, and Cd exposure.

The genes that had the most significant expressed on *Z. japonica* were first identified. Therefore, genes that were co-expressed across different treatments may exhibit highest maximum likelihood values. Co-regulators associated with expression differed among all three metal treatments, as is shown in the Venn diagram (Figure 2A), and the overlapping regions (2001 individual genes) represent DEGs that were common in multiple treatments. The co-expressed genes were further clustered using DAVID functional annotation clustering at the highest stringency classification (Figure 5A). These process groups consisted of biological processes that have similar biological meaning because of shared genes. The results identified co-expressed genes that were associated with metal ion binding, response to chemical stimulus and wounding, photosynthesis, oxidoreductase activity, and peroxidase activity in molecular function, the transmission of messages between cells, and metabolic process, electron transport biological process (Figure 4D). The annotations of common genes in these blocks underwent NCBI non-redundant protein sequences (NR) database analyses and the most closely related species *Zostera marina* database (Table 2), and hits with maximum alignment lengths and sequence identity were identified as probable orthologs.

**Table 2:** List of co-expressed genes from the *Zostera marina* database and NR database.

**Unigenes**	**Gene ID (*Zostera marina*)**	**Description (*Zostera marina*)**	**NR ID**	**NR description**
c6564_g2	ZOSMA_15G00610	Isoflavone reductase-like protein	XP_010940090.1	PREDICTED: isoflavone reductase-like protein isoform X1 [Elaeis guineensis]
c42084_g1	ZOSMA_271G00210	Chitinase, family GH18	XP_003564668.1	PREDICTED: hevamine-A-like [Brachypodium distachyon]
c33717_g1	ZOSMA_10G00200	40S ribosomal protein S2-4	OAY48510.1	Hypothetical protein MANES_06G163300 [Manihot esculenta]
c42083_g1	ZOSMA_32G00340	Uncharacterized protein	XP_006287545.1	Hypothetical protein CARUB_v10000754mg [Capsella rubella]
c57819_g2	ZOSMA_45G00680	Putative Cinnamoyl-CoA reductase	XP_010932148.1	PREDICTED: cinnamoyl-CoA reductase 1-like [Elaeis guineensis]
c27603_g1	ZOSMA_81G00380	Putative Pentatricopeptide repeat-containing protein	XP_006645835.1	PREDICTED: peroxidase 1-like [Oryza brachyantha]
c53035_g1	ZOSMA_8G01400	Harpin-induced like protein 3	XP_006854681.1	PREDICTED: protein YLS9 [Amborella trichopoda]
c63283_g1	–	–	XP_002315724.2	Trypsin inhibitor family protein [Populus trichocarpa]
c21323_g2	–	–	XP_007217744.1	Hypothetical protein PRUPE_ppa008516mg [Prunus persica]
c24262_g1	ZOSMA_187G00150	–	NP_001280836.1	Granule-bound starch synthase 1, chloroplastic/amyloplastic-like [Malus domestica]
c2586_g1	ZOSMA_121G00350	ABC transporter G family member	XP_016541771.1	PREDICTED: ABC transporter G family member 9-like isoform X1 [Capsicum annuum]
c41999_g1	ZOSMA_182G00410	ABC transporter B family member 11	KMZ71350.1	ABC transporter B family member 11 [Zostera marina]
c4316_g1	–	–	KMZ61391.1	Hypothetical protein ZOSMA_52G00420 [Zostera marina]
c67436_g1	ZOSMA_40G00520	Long-Chain Acyl-CoA Synthetase	KMZ72313.1	Long-Chain Acyl-CoA Synthetase [Zostera marina]
c6558_g1	–		XP_002951489.1	hypothetical protein VOLCADRAFT_92044 [Volvox carteri f. nagariensis]
c37417_g1	–	–	AAF21988.2AF116537_1	Fiber protein GLP1 [Gossypium hirsutum]

Furthermore, most of genes reflect the cellular response to stimulus, certain key gene regulators such as c6564_g2 response to oxidative stress, c53035_g1 and c63283_g1 response to chemical stimulus and wounding, c37417_g1 about nutrient reservoir activity, c21323_g2 in peroxidase activity and c57819_g2 which is involved in oxidoreductase activity were significantly highly expressed of following exposure to the three metals (*padj* < 0.05). More than that, genes such as c42083_g1 in the electron transport, c41999_g1 *ABCs* signal transduction, c6558_g1 and c2586_g1 in photosynthesis, light reaction and *MT*_*S*_ were significantly upregulated or downregulated following both three exposure (Figure 5A and Table 2). In higher plants, MT induction seems to be metal-specific. Plant MT genes were proved to be induced by many abiotic and biotic effectors, and play important roles in maintenance of homeostasis related to essential metal transition, detoxification of toxic metals, and protection against oxidative stress (Zimeri et al., 2005). In *P. oceanica*, MT mRNAs were increased by Cu and Cd, but not by Hg (Giordani et al., 2000), and this result is similar to what we observed in *Z. japonica* plants that were exposed Cu, Cd, and Pb groups. Genes including *PSBS, PSAG, psaJ, psbA, psbD*, etc. (Dattolo et al., 2014) are usually recognized as photosynthetic response genes, and they were detected in this study at significant expression levels. Heat shock proteins (*HSPs*) are often employed as a primary response to dampen heat stress effects, and play important roles in seagrass adaptability and resilience and (Gu et al., 2012; Franssen et al., 2014), our study also found *HSP20* gene expression in all three metal exposure treatments. Therefore, it can be speculated that *HSP* genes are sensitive to some abiotic stresses. Because most of the non-model organisms bioinformatics resources for seagrasses are limited. There is a need for user-friendly and interactive web portals, whereby the user can perform annotation and mine for candidate genes, as well as genetic markers. Thus, sequencing of many more seagrass species like *Z. japonica* is necessary.

Changes in the regulation of a detoxification process including upregulation of genes coding detoxifying enzymes, such as cytochrome P450, UDP-glucose transferase, the ABC-transporter, as well as *HSP*s as a stress response gene to heavy metal (copper, Cu) was first shown in the seaweeds species Laminaria digitata and E. siliculosus (Ritter et al., 2008, 2014). In seagrasses, oxidative stress-protective genes have oftern been recognized and associated with light response (Dattolo et al., 2013), thermal stress (Franssen et al., 2011, 2014; Winters et al., 2011) and extreme environments (Lauritano et al., 2015). While the catalase gene has been reduced to a single copy in *Z. marina* (possibly due to reduced xylemcharacteristics of submerged plants), whilst all three types of superoxide dismutase remain (Olsen et al., 2016). Glutathione-related transcripts have been isolated and profiled in *P. oceanica, Z. muelleri*, and *Z. marina* (Massa et al., 2011; Lauritano et al., 2015; Olsen et al., 2016; Pernice et al., 2016). In protein, enzymes related to cellular stress and photosynthesis genes in which the studies of seagrass in response to differing light conditions also revealed the differential expression of genes for (Dattolo et al., 2013, 2014).

We also found a number of genes that were uniquely expressed in each metal (Datasheets S1–S4, Figure 5B). Genes for c21603_g1 about transposase, c35877_g1 in the Phenylpropanoid biosynthesis are up- regulated, *c31516_g1* in integral to membrane, and *c40473_g1* in the proteolysis and metabolic process were down-regulated and all of them are uniquely expressed in Cd treatment plants. Plants from the Pb treatment expressed genes are more than Cd and Cu treatment, such as chromatin-remodeling complex ATPase ISWI2(ISS)( c36097_g2) in nucleus position which is responsible for chromatin remodeling function, c32733_g1 which engaged in aminoacyl-tRNA biosynthesis, transmembrane transport genes (c34886_g2), c36004_g2 acted as transferase activity, c36201_g2 in protein kinase PCTAIRE and related kinases are all highly up-regulated.Similarly, genes associated with c38267_g3 in Cu ion transport process, c34401_g1 about Aryl sulfotransferase, c32383_g1 in Ubiquitin-conjugating enzyme and c33655_g1 in premnaspirodiene oxygenase were specifically expressed in the Cu treatment group.

## Conclusion

*Zostera japonica* accumulated higher Cu, and the genes like c34401_g1 about Aryl sulfotransferase, c32383_g1 in Ubiquitin-conjugating enzyme and c33655_g1 in premnaspirodiene oxygenase were highly expressed. RNA-seq technology was employed to obtain a genome-wide account of the transcriptional responses to Cu, Pb, and Cd stress in intertidal seagrass *Z. japonica*. Expanding on previous field investigations and laboratory work, 2001 target co-expressed genes associated with metal transcriptional regulation were identified. Our results highlighted an important role of the co-expressed genes for c6558_g1 about ion binding, c6564_g2 in oxidoreductase activity, c37417_g1 in nutrient reservoir activity were highly expressed, and upregulated, which can be regarded as indicator gene for heavy metal Cu, Pb, and Cd stress plants. There is no doubt that transcriptional metal stress responses are dynamic over time. However, this genome-wide approach included only a single time point of Cu, Pb, and Cd exposure. We observed a substantial degree of qualitative consistency between the responses to the different growth systems used in this and previous studies (unpublished data), which span a wide range of plant ages and different levels of Cu, Pb, and Cd exposure. Therefore, we can conclude that this study captured a large number of transcriptional responses that are of fundamental and general importance. Molecular studies have provided accurate insight into the role of associated transcription regulators in seagrasses. In seagrasses studies, transcriptome have provided us with snapshots of gene expression at given times under specific conditions in species. Our understanding of molecular and functional diversity will also help redefine our understanding of ecological concepts associated with the adaptability of seagrass, especially with regard to this endangered species in Asia. Most of studies have focused on short-term response, so, there is a need for validated molecular markers are the requisite given the growing number of pressures which seagrass meadows are faced with.

## Author contributions

HL, YZ, and TS wrote the main manuscript text. RG, XZ, and HL did the RT-qPCR validation, bioinformatics analysis by HL, WY, and Allwegene Bioinformatics Technology team. All authors reviewed the manuscript.

### Conflict of interest statement

The authors declare that the research was conducted in the absence of any commercial or financial relationships that could be construed as a potential conflict of interest.
