# Supplementary material for: Which Genes in a Typical Intertidal Seagrass (Zostera japonica) Indicate Copper-, Lead-, and Cadmium Pollution?
Source: Front Plant Sci. 2018 Oct 24;9:1545. doi: 10.3389/fpls.2018.01545 (PMC6207952; doi:10.3389/fpls.2018.01545)
Supplement: Supplementary file 1 [file Presentation_1.pdf]

Which genes in a typical intertidal seagrass (*Zostera japonica*) indicate Copper-, lead-, and cadmium pollution?

Haiying Lin[1], Tao Sun[1,*], Yi Zhou[2,**], Ruiting Gu[2], Xiaomei Zhang[2,†], and Wei Yang[1,†]

[1]State Key Laboratory of Water Environment Simulation, School of Environment, Beijing Normal University, Beijing 100875, PR China

[2]Key Laboratory of Marine Ecology and Environmental Sciences, Institute of Oceanology, Chinese Academy of Sciences, Qingdao 266071, PR China

[*]Corresponding author: Tel & fax: 86-10-58805053.E-mail address: suntao@bnu.edu.cn (T. Sun).

[**]Corresponding author: Tel & fax: 86-532-82898646. E-mail address: yizhou@qdio.ac.cn (Y. Zhou).

†these authors contributed equally to this work

SUPPORTING INFORMATION (SI)

Additional supporting information may be found in the online version of this article.

1st: SI in supplementary.

Table S1. Translocation factor (TF) of *Zostera japonica* tissues under different metal exposure experiments over a seven-day period.

Table S2.The quality of the raw reads.

Figure S1. Transcript length distribution.

Figure S2. A) Differentially expressed gene levels for each treatment. B) Density distribution of FPKM used to test gene expression profiles of different heavy metal treatment samples.

Table S3. Number of upregulated and downregulated genes in Zostera japonica tissues under different metal exposure treatments.

Fig. S3. Differentially expressed genes (DEGs) in control and metal treatments. The "padj < 0.05" criterion was used as the threshold to determine the significance of gene expression differences. Hierarchical clustering (padj < 0.05) of the differentially expressed genes in *Z. japonica* exposed to Cu, Pb, and Cd is shown. Each row represents a single gene, and each column represents one metal treatment.

Table S4. Differentially expressed transcripts from analyzed data and primers for RT-qPCR analyses.

Figure S4. Gene annotation success rate statistics.

Table S1. Translocation factor (TF) of *Zostera japonica* tissues under different metal exposure experiments over a seven-day period.

	0.5 d	1 d	2 d	4 d	7 d
TF-Cu	1.25	1.19	1.51	2.39	1.55
TF-Pb	0.91	1.03	0.85	0.67	0.68
TF-Cd	1.05	0.99	0.84	0.72	0.98

Table S2. The quality of the raw reads.

Sample	Raw Reads	Clean reads	Clean bases	Error (%)	Q20 (%)	Q30 (%)	GC (%)
Control1	25694356	25284504	3.8	0.02%	97.85%	94.60%	46.78%
Control2	24328110	23906340	3.58	0.02%	97.90%	94.72%	46.47%
Control3	26788360	26302750	3.94	0.02%	97.92%	94.77%	46.67%
Cu_7d_1	23458620	23091126	3.46	0.02%	97.73%	94.37%	46.78%
Cu_7d_2	24339176	23925316	3.58	0.02%	97.93%	94.79%	46.67%
Cu_7d_3	26152950	25748080	3.86	0.02%	97.94%	94.81%	46.74%
Pb_7d_1	25858496	25085318	3.76	0.01%	98.11%	95.19%	46.61%
Pb_7d_2	26028802	25388840	3.8	0.02%	98.00%	94.93%	46.92%
Pb_7d_3	23433144	22833274	3.42	0.02%	98.03%	94.98%	46.91%
Cd_7d_1	22878208	22432100	3.36	0.02%	97.94%	94.81%	46.22%
Cd_7d_2	22681514	22278756	3.34	0.02%	98.00%	94.94%	46.31%
Cd_7d_3	23474758	23099814	3.46	0.02%	97.92%	94.77%	46.28%

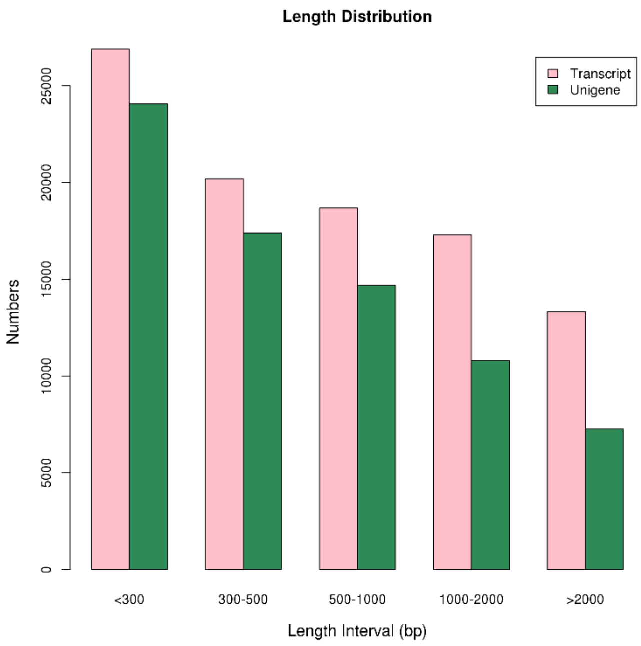

Figure S1. Transcript length distribution.

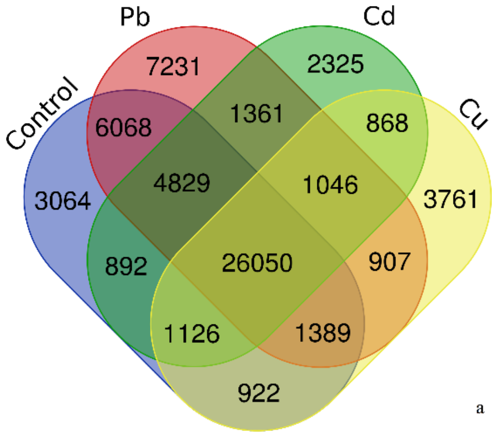

a

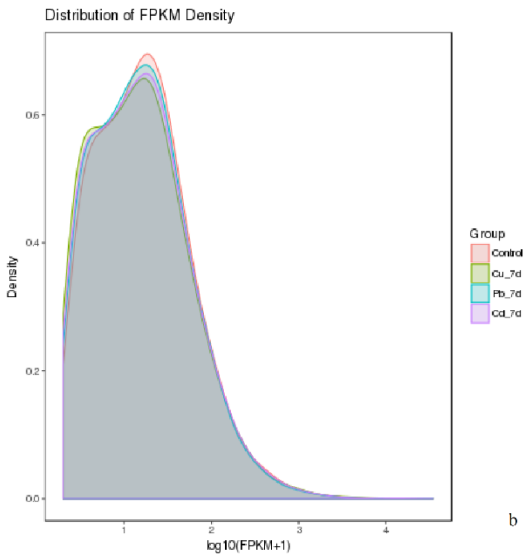

b

Fig. S2. a) Differentially expressed gene levels for each treatment. b) Density distribution of FPKM used to test gene expression profiles of different heavy metal treatment samples.

Table S3. Number of upregulated and downregulated genes in *Zostera japonica* tissues under different metal exposure treatments.

	Upregulated genes			Downregulated genes		
Single	Cu	Pb	Cd	Cu	Pb	Cd
	1700	4132	740	1011	1252	558
2 metals	Cu & Pb	Pb & Cd	Cu & Cd	Cu & Pb	Pb & Cd	Cu & Cd
	70	152	1121	462	407	1062
3 metals	Cu, Pb, & Cd			Cu, Pb, & Cd		
	375			400		

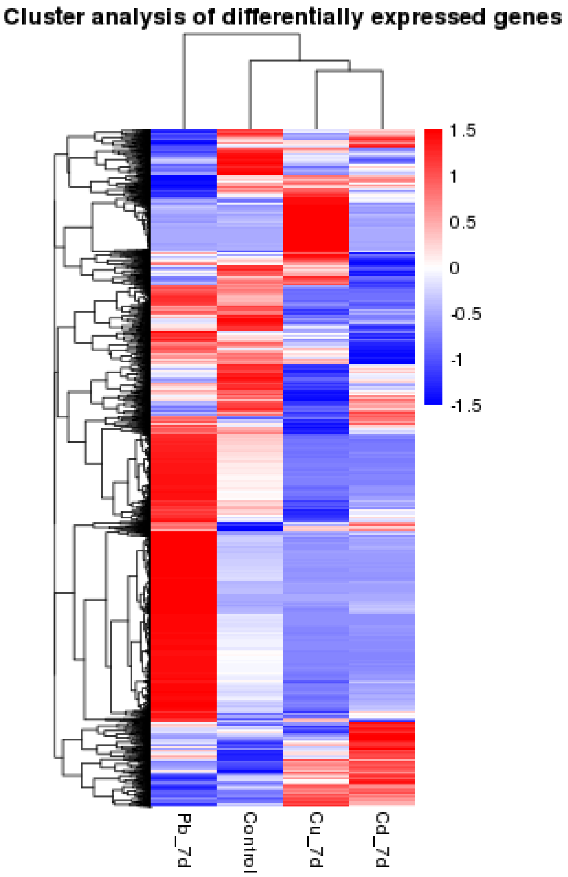

Fig. S3. Differentially expressed genes (DEGs) in control and metal treatments. The "padj < 0.05" criterion was used as the threshold to determine the significance of gene expression differences. Hierarchical clustering (padj < 0.05) of the differentially expressed genes in *Z. japonica* exposed to Cu, Pb, and Cd is shown. Each row represents a single gene, and each column represents one metal treatment.

Table S4. Differentially expressed transcripts from analyzed data and primers for RT-qPCR analyses.

	Gene name	Treatment	Annotation	Expression	Primers	
Specific-gene (8 items)	c41999_g1	Cu	multidrug resistant ABC transporter family protein [Populus trichocarpa]	down	c41999-F<br>c41999-R	TGCTTCTGCAATAATGTCCAG<br>GTTGGTTATATCTATCAACAG
	c20091_g1	Cu	DNA binding protein, putative [*Ricinus communis*]	up	c20091-F<br>c20091-R	ACAGCAGAGGATGCCGCACG<br>TCCAGTCGAGGGTCGCGGAG
	c40663_g1	Cu	alpha carbonic anhydrase 7-like [Musa acuminata subsp. malaccensis]	down	c40663-F<br>c40663-R	CACTATGATCAACCGAGGTC<br>ACCATGTGCATCTCCATGTC
	c14893_g2	Cd	polygalacturonase [*Elaeis guineensis*]	down	c14893-F<br>c14893-R	AGAGTCGCATGGTAACGATC<br>GTTGCTACTAGATTCCTTGC
	c67519_g1	Cd	zeaxanthin epoxidase [*Scutellaria baicalensis*]	down	c67519-F<br>c67519-R	CAGTGACGAGAGTGATAAGTC<br>ATCGCCTTCATGCTGTTCTC
	c24262_g1	Pb	granule-bound starch synthase 1, chloroplastic/amyloplastic [Phoenix dactylifera]	up	c24262-F<br>c24262-R	AACTGGAGGACTTGGTGATG<br>ACGAACAGTATAGAGCTTGT
	c47806_g1	Pb	[starch synthase 1, chloroplastic/amyloplastic-like [*Citrus sinensis*]	up	c47806-F<br>c47806-R	ATGTCGTAGTTCACCTCAGTG<br>GAGTATGGTGCGACCTCAGCG
	c58282_g1	Pb	glucose-6-phosphate/phosphate translocator 2, chloroplastic-like [*Musa acuminata subsp. malaccensis*]	up	c58282-F<br>c58282-R	GCTTGAAGTCGTCTCACGGA<br>GTTGTAGATATTGAACACGAC
Two overlap (3 items)	c41999_g1	Cd + Cu	multidrug resistant ABC transporter family protein [*Populus trichocarpa*]	down	c41999-F<br>c41999-R	TGCTTCTGCAATAATGTCCAG<br>GTTGGTTATATCTATCAACAG
	c14776_g1	Cd + Cu	vacuolar amino acid transporter 1 [*Musa acuminata* subsp. *malaccensis*]	up	c14776-F<br>c14776-R	CTACACGATATCTCTCAACG<br>GCCACAAGCTCGGCAGTGCT
	c14476_g1	Cd + Pb	copia-like retrotransposon Hopscotch polyprotein [*Zea mays*]	down	c14476-F<br>c14476-R	ATGATGAGGTCATGATTGGT<br>GTAAATTGTAATGATGAACCA
Three overlap (1 items)	c39244_g1	Cu + Cd + Pb	metalloendoproteinase 1 precursor [Zea mays]	down	c39244-F<br>c39244-R	ATGGTGTGCTAGATGCAGCC<br>GTCGCTAGTATAGTGGTATCT
			Reference genes		Zostera-18S-F<br>Zostera-18S-R	TCGGACGGTTTTGTGGTGA<br>CCTTCCTTGGATGTGGTAGCC

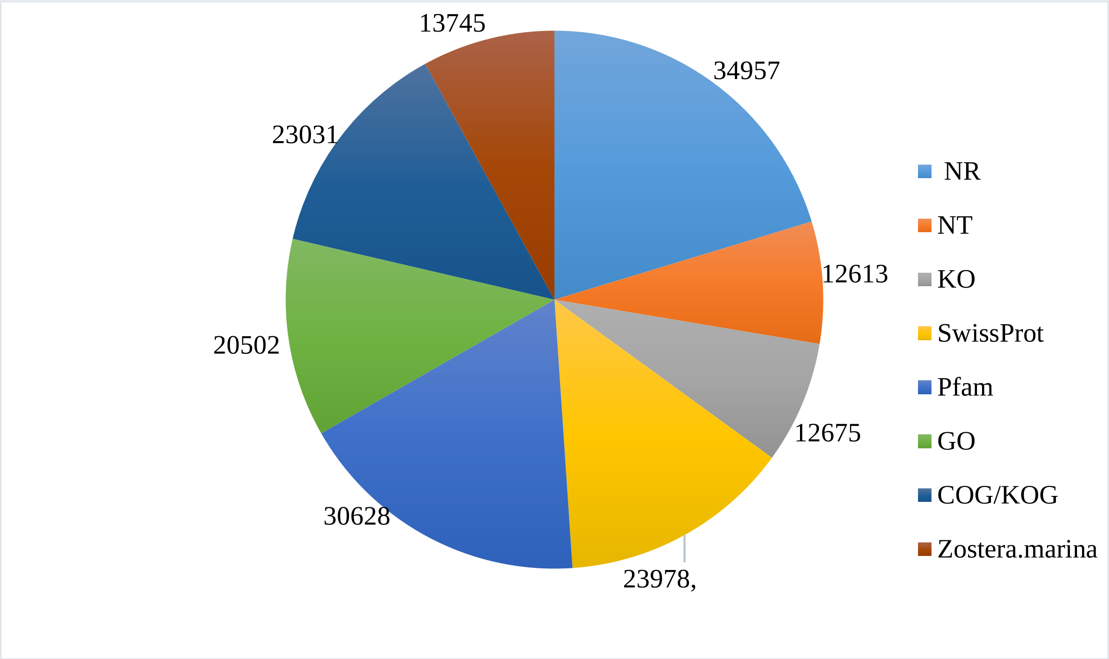

Figure S4. Gene annotation success rate statistics.
